# Effect of feeding a dried distillers’ grains with solubles diet on the metabolism of the intestinal wall in Guanling crossbred cattle: a preliminary assessment

**DOI:** 10.3389/fvets.2023.1223088

**Published:** 2024-01-09

**Authors:** Xiaofen Luo, Tiantian Zhang, Duhan Xu, Mingming Zhu, Junjie Zhang, Rong Zhang, Guangxia He, Ze Chen, Shihui Mei, Bijun Zhou, Kaigong Wang, Chao Chen, Erpeng Zhu, Zhentao Cheng

**Affiliations:** ^1^College of Animal Science, Guizhou University, Guiyang, China; ^2^Key Laboratory of Animal Diseases and Veterinary Public Health of Guizhou Province, College of Animal Science, Guizhou University, Guiyang, China

**Keywords:** dried distillers’ grains with solubles (DDGS), Guanling crossbred cattle, intestinal wall, metabolite, metabolic pathways

## Abstract

Dried distillers’ grains with solubles (DDGS)-based diets are nutritious and can improve the inflammations and intestinal immunity in livestock. However, there is limited research examining the effect of feeding DDGS-based diets on changes in intestinal metabolites and related pathways in livestock. In this study, six Guanling crossbred cattle (Guizhou Guanling Yellow cattle × Simmental cattle) were selected and divided into a basal diet (BD) group and an experimental group fed with DDGS replacing 25% of the daily ration concentrates (DDGS) (*n*=3), respectively. Fresh jejunum (J), ileum (I) and cecum (C) tissues were collected for metabolomic analysis. Differential metabolites and metabolic pathways were explored by means of univariate and multivariate statistical analysis. In comparison to the J-BD group, 123 differential metabolites (VIP > 1, *p* < 0.05) were identified in the J-DDGS group, which (top 20) were mainly divided into superclasses, including lipids and lipid-like molecules, organic acids and derivatives, and organoheterocyclic compounds. Compared with the I-BD group, 47 differential metabolites were obtained in the I-DDGS group, which were mainly divided into superclasses, including lipids and lipid-like molecules and organic acids and derivatives. The C-DDGS vs. C-BD comparison revealed 88 differential metabolites, which were mainly divided into superclasses, including lipids and lipid-like molecules, organic oxygen compounds, and nucleosides. A total of 34 significant metabolic pathways were found (*p* < 0.05, −log(*p*) > 1.3). Among them, 3 significant pathways were significantly enriched in the J-DDGS group, 11 significant pathways were significantly enriched in the I-DDGS group, and 20 significant pathways were significantly enriched in the C-DDGS group. Importantly, primary bile acid biosynthesis, linoleic acid metabolism, and arachidonic acid metabolism correlated with intestinal inflammation and immunity by regulating gut microbiota, prostaglandin synthesis, and cell signaling. The data suggest that DDGS-fed cattle unregulated three metabolic pathways mentioned above and that a DDGS-based diet was able to maintain a balance of these three metabolic pathways, thus resulting in improvement of intestinal inflammation and enhanced immunity in cattle. In conclusion, the DDGS diet has the potential to improve intestinal inflammation and enhance the immunity of Guanling crossbred cattle by regulating the metabolic patterns of lipids and lipid-like molecules, organic acids and derivatives, and related metabolic pathways. These results allude to potential metabolic regulatory mechanisms of DDGS diets and also provide a theoretical basis for the application of DDGS in livestock feed.

## 1 Introduction

The liquor industry is traditional in China and is also an important economic industry in Guizhou province. Chinese liquor production reached 7.859 million kiloliters in 2019 and produced large numbers of distillers’ grains (DGs) (1). DGs are produced from sorghum, corn, barley, and other pure grains. DGs contain a lot of protein, fat, cellulose, yeast, and enzymes, which are hard for animals to digest and absorb completely. Fresh DGs are perishable (2), and fresh DGs fed directly can easily cause acidosis (3). Therefore, drying fresh DGs to obtain dried distillers’ grains with solubles (DDGS) not only stabilizes the protein content and retains vitamins but also improves the palatability of the feed, prevents mold and acidity from affecting the odor and taste, extends the shelf life, widens the application range and improves the application effect of DGs in animal husbandry (1). Feeding dried DGs has been evidenced to improve the growth performance and meat quality of bulls (4). Current studies on the utilization of DDGS as animal feeds focus on the improvement of animal growth performance, weight gain, milk production in dairy cows, meat quality in beef cattle, antioxidant capacity, and stress reduction (5–9).

The intestine is an important immune-digestive organ of ruminants and is one of the habitats for a large number of microorganisms that play an important role in the energy metabolism of animals and also produce metabolites in direct contact with cells of the intestinal wall (10). The intricate relationship between metabolism and the regulation of immunity and inflammation has been increasingly highlighted in research (11–13). Inflammation is a significant concern in cattle intestines as it disrupts the normal functioning of the intestinal barrier, compromises nutrient absorption, and alters immune homeostasis in cattle (14). Metabolites, which are the small molecules produced during metabolism, have emerged as important mediators in modulating immune responses and inflammatory processes in various organisms (15). Whilst it has been confirmed that these metabolites, resolvins, protectins, and maresins, are generated endogenously by the host, there is an escalating body of evidence that demonstrates intestinal metabolism may produce exclusive mediators that hold biological activity. Certain intestinal metabolites, including essential polyunsaturated fatty acids (PUFAs) such as ω3 and ω6, have been found to significantly impact the host immune system (16). In particular, lipid metabolites derived from ω3-PUFA, such as eicosapentaenoic acid and docosahexaenoic acid, have recently demonstrated potent anti-inflammatory and antiallergenic properties (17). To date, the majority of the studies mentioned have concentrated on regulating the structure and metabolism of intestinal flora in the intestinal contents, with minimal consideration for intestinal wall metabolism. The alteration of indicators in intestinal contents represents, to some extent, the combined effects of bodily function and intestinal microorganisms. However, the metabolic profile of the intestinal wall tends to lean toward signaling alterations in bodily metabolism. We conducted a study to examine the impact of feeding DDGS diets on the intestinal metabolic profile in Guanling crossbred cattle using LC–MS-based non-targeted metabolomic techniques. Our findings offer novel insights into the regulatory effects of dietary DDGS supplementation on intestinal metabolites and their metabolic pathways in ruminants, which could facilitate the future development and utilization of DDGS as a potentially valuable feed resource.

## 2 Materials and methods

### 2.1 Origin and treatment of DDGS

The DDGS used in this study were obtained from the Kweichow Moutai Group in Moutai Town, Renhuai, Guizhou, China. The main ingredients of Moutai DGs are distilled sorghum and wheat, which are byproducts of the brewing processes. A drum dryer was used for the drying treatment of the DGs. After centrifugation of fresh Moutai DGs, the filter residues and filtrate were separated, and the filtrate was evaporated and concentrated, and then mixed and dried together with the filter residues to obtain DDGS with a moisture content of 10**–**15%.

### 2.2 Animal experiment

This study was conducted at the Yueyawan cattle farm in Guanling County, Anshun City, Guizhou Province (105^°^ 58′ E, 25^°^ 98′ N, altitude, approximately 1863 m). The experimental period was from September 2020 to November 2020, and the average outdoor minimum temperature was 16.9°C and the maximum temperature was 25.6°C. Six Guanling crossbred cattle (Guizhou Guanling Yellow cattle × Simmental cattle) in good health and of similar age (18 months old), weighing 456 ± 26 kg, were provided by the beef cattle fattening farm of Guanling County Yellow Cattle Group. Laboratory tests showed that these cattle were negative for *brucella*, *mycobacterium tuberculosis*, foot-and-mouth disease virus, and lumpy skin disease virus infection. Two experimental groups (containing three cattle each) were randomly assigned to one of the two dietary treatments: a basal diet (BD) and a DDGS diet (Jiang-flavor DDGS replaces 25% of the concentrate). The BD was formulated based on the nutritional requirement of 300 kg body weight and 1 kg/d average daily gain according to China’s Beef Feeding Standard (NY/T815-2004), with a forage:concentrate ratio of 60:40 on a dry matter basis. The dietary composition and nutrient levels are shown in Supplementary Tables S1, S2 (17). The cattle were fed at 9:00 and 16:30 every day for 75 days (15 days for adaptive feeding and 60 days for formal feeding of the experimental diet). During the experiment, the cattle drank water freely, and hygiene and daily management were carried out as a matter of routine.

### 2.3 Collection and preparation of samples

On the 60th day of the formal feeding period, the animals underwent overnight fasting, and then tissues were collected from the jejunum, ileum and cecum, namely, jejunum-BD (J-BD), jejunum-DDGS (J-DDGS), ileum-BD (I-BD), ileum-DDGS (I-DDGS), cecum-BD (C-BD), and cecum-DDGS (C-DDGS). The intestinal sample (intestinal wall) collected in this study refers to the entire thickness of the intestine anatomically including the mucosal layer, submucosal layer, muscle layer, and serosal layer; and it was washed with sterile saline to remove the internal contents. The quality control (QC) samples were obtained by mixing equal volumes of the extracts from all collected samples. Prior to the metabolomics analysis, 30 mg of jejunum, ileum, and cecum tissues were weighed using a JC-TP1203 One Thousandth Electronic Balance (Jingcheng Instrument, Qingdao, China) and transferred into a 1.5-mL tube with two small steel balls. We regarded 20 μL of L-2-chlorophenylalanine (0.3 mg/mL, Hengchuang Biotechnology Co, Shanghai, China) dissolved in methanol (Amperexperiment Technology Co, shanghai, China) as an internal standard. Then 400 μL mixture of methanol/water (1/4, v/v, Amperexperiment Technology Co, Shanghai, China) was added and pre-chilled at −20°C for 2 min and then ground in a grinder for 2 min. The mixtures were subject to ultrasonic separation for 10 min, stored at −20°C for 30 min, and then centrifuged at 13,000 × *g* for 10 min at 4°C. A total of 300 μL of supernatant was dried in a freeze-concentration centrifugal dryer and resolubilized by 200 μL methanol/water (1/4), then vortexed for 30 s and extracted by ultrasonic for 3 min. After placement for 2 h at −20°C, samples were centrifuged at 4°C (13,000 × *g*) for 10 min, and 150 μL supernatants were filtered through 0.22 μm microfilters and then transferred to liquid chromatography (LC) vials (18). All chemicals and solvents are analytically pure or of chromatographic grade.

### 2.4 LC–MS analysis

The LC–MS analysis of jejunum, ileum, and cecum samples in both ESI positive and ESI negative ion modes was performed using the ACQUITY UPLC I Class system (Waters Corporation Milford, USA) coupled with VION IMS QTOF mass spectrometer (Waters Corporation Milford, USA). A 1 μL aliquot of each intestinal tissue sample was injected onto a column ACQUITY UPLC™ BEH C18 (50 mm × 2.1 mm i.d.,1.7 μm; Waters Corporation, Milford, MA, USA). Mobile phase A was 0.1% formic acid in water, and phase B was an acetonitrile/methanol solution (2/3) containing 0.1% formic acid (v/v); the flow rate was 0.4 mL/min. The conditions for UPLC separation and ESI-VIONIMS Q-TOF detection are shown in Supplementary Tables S3, S4. The QC samples were used to evaluate the reproducibility and reliability of the LC–MS system, which contains metabolic information that enables the assessment of the stability of the mass spectrometry system. The ion peaks with a relative standard deviation of >0.4 for the QC group samples were removed, and the stability of the system was evaluated by principal component analysis (PCA). The PCA model plot was obtained after 7 cycles of cross-validation to detect whether the QC samples were closely clustered together, thus judging the stability of the instrument’s detection.

### 2.5 Data analysis

The original data were processed by the Progenesis QI v2.3 (Nonlinear Dynamics, Newcastle, UK), and metabolites were qualitatively analyzed using the Human Metabolome Database (HMDB), Lipid maps (v2.3), the METLIN database, and a self-created database. For the obtained data, the ion peaks with >50% missing values (0 value) in this group, replacing the 0 value with half of the minimum value, were screened to qualitatively obtain compounds according to the qualitative result evaluation (score). The qualitative results were screened between 36 and 60 points, below which they were considered inaccurate and deleted. Finally, the positive and negative ion data were combined into a data matrix containing all the information extracted from the original data necessary for further analysis. Subsequently, multivariate statistical analyses were carried out using SIMCA 14.0 (Umetrics AB, Umeå, Sweden).

The PCA and orthogonal partial least squares discriminant analysis (OPLS-DA) were performed to reveal the global metabolic changes between the BD and DDGS groups using SIMCA 14.0. An unsupervised PCA was first used to examine both the metabolic differences between the groups and the individual metabolic differences within samples. To maximize the differences between groups within the model, a supervised OPLS-DA was performed on metabolites between groups. In addition, to prevent overfitting of the model, the quality of the model was examined by response permutation testing with 200 responses. Then, univariate analyses were performed on the sample data, which consisted of the student’s *t*-test and fold change analysis. The fold change (FC) value was calculated through a fold analysis of variance to assess differences in metabolite expression between any two groups. The value of *p*s were obtained using a student’s *t*-test, and the volcano plotting was produced using Origin 2020 (Origin Lab, Northampton, MA, USA) to visualize the value of *p*s and FC values to facilitate rapid identification of metabolites with significant differences. Multivariate and univariate statistical analyses were combined to screen the differential metabolites between groups.

### 2.6 Key biomarkers and metabolic pathways analysis

To reveal the mechanism of metabolic pathway variation in intestinal samples, an analysis of differential metabolites was carried out with metabolic pathway enrichment analysis based on the Kyoto Encyclopedia of Genes and Genomes (KEGG) database.^1^ Their KEGG IDs and pathways were obtained, and then the number of metabolites enriched in each corresponding pathway was calculated. The pathway with a value of *p* < 0.05 was selected as an enriched pathway. The formula for calculating value of *p*s was as follows:


$$P=1-\overset{m-1}{\underset{i=0}{\sum }}\frac{\left(\begin{array}{c} M\\ i \end{array}\right)\left(\begin{array}{c} N-M\\ n-i \end{array}\right)}{\left(\begin{array}{c} N\\ n \end{array}\right)}$$

where N is the total number of metabolites, n is the number of differential metabolites, M is the number of metabolites annotated as a specific pathway, and m is the number of differential metabolites annotated as a specific pathway. A smaller value of *p* indicates a more pronounced difference within that metabolic pathway.

### 2.7 Statistical analyses

All continuous variables were expressed as the mean ± standard deviation (M ± SD). The differences between the groups were analyzed using a student’s *t*-test, where a significant difference was set at *p* < 0.05. All statistical analyses were performed using SPSS Statistics 26.0 (Chicago, IL, USA).

## 3 Results

### 3.1 Quality control of the metabolomic data of the intestinal tissues

**Figure 1:**
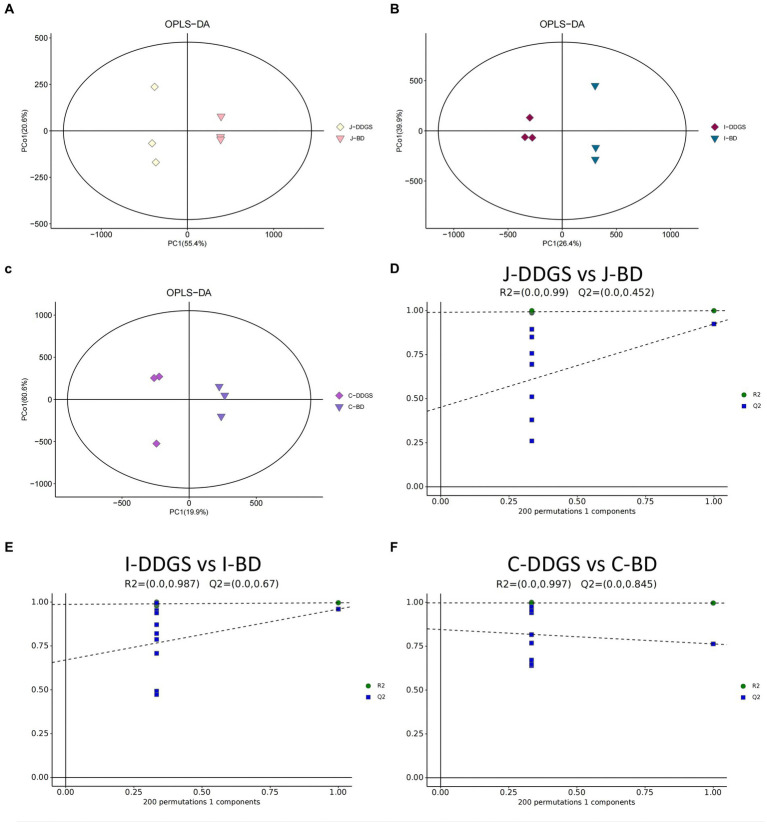
OPLS-DA for data distribution. 2D score scatter plot of the PCA model for the **(A)** J-DDGS vs. J-BD, **(B)** I-DDGS vs. I-BD, and **(C)** C-DDGS vs. C-BD comparisons, respectively. Permutation plots for the OPLS-DA model show R2 (blue) and Q2 (green) values. The results of the permutation test strongly indicate that the original model was valid **(D–F)**. Each data point represents one sample of intestinal tissue, and two coordinate points relatively far apart on the score plot indicate a significant difference between the two samples, and vice versa. The oval area indicates the 95% confidence interval. BD, basal diet group, DDGS: mixed diet group containing 25% DDGS derived from Moutai-flavored DG.

LC–MS full-scan detection method was used to illustrate the metabolic alterations in the jejunum, ileum, and cecum tissues from Guanling crossbred cattle fed DDGS diets. A total of 11,028 substance peaks, including 8,464 substance peaks in ESI^+^ mode and 2,564 substance peaks in ESI^−^ mode, were detectable, and a total of 1,399 metabolites were identified. PCA scoring plots were initially generated using the processed data obtained from metabolomic analysis of the jejunum, ileum, and cecum tissues to evaluate systematic errors in the samples and the trends within each comparison. As shown in Supplementary Figure S1A, the quality control (QC) samples clustered in the center of the PCA score plots, suggesting that the analyses were repeatable and stable. Clear separation trends were observable in the J-DDGS vs. J-BD, I-DDGS vs. I-BD, and C-DDGS vs. C-BD comparisons (Supplementary Figures S1B–D). A tendency for separation on the metabolic spectrum in J-DDGS vs. J-BD, I-DDGS vs. I-BD, and C-DDGS vs. C-BD comparisons was observed in the OPLS-DA score chart (Figures 1A–C). The results of the permutation test strongly indicated that the original model was valid (J-DDGS vs. J-BD: R^2^ intercept = 0.990, Q^2^ intercept = 0.452; I-DDGS vs. I-BD: R^2^ intercept = 0.987, Q^2^ intercept = 0.670; C-DDGS vs. C-BD: R^2^ intercept = 0.997, Q^2^ intercept = 0.845, Figures 1D–F). These results provide assurance for subsequent tests and analyses.

### 3.2 Differential metabolites in intestinal tissues of DDGS and BD groups

**Figure 2:**
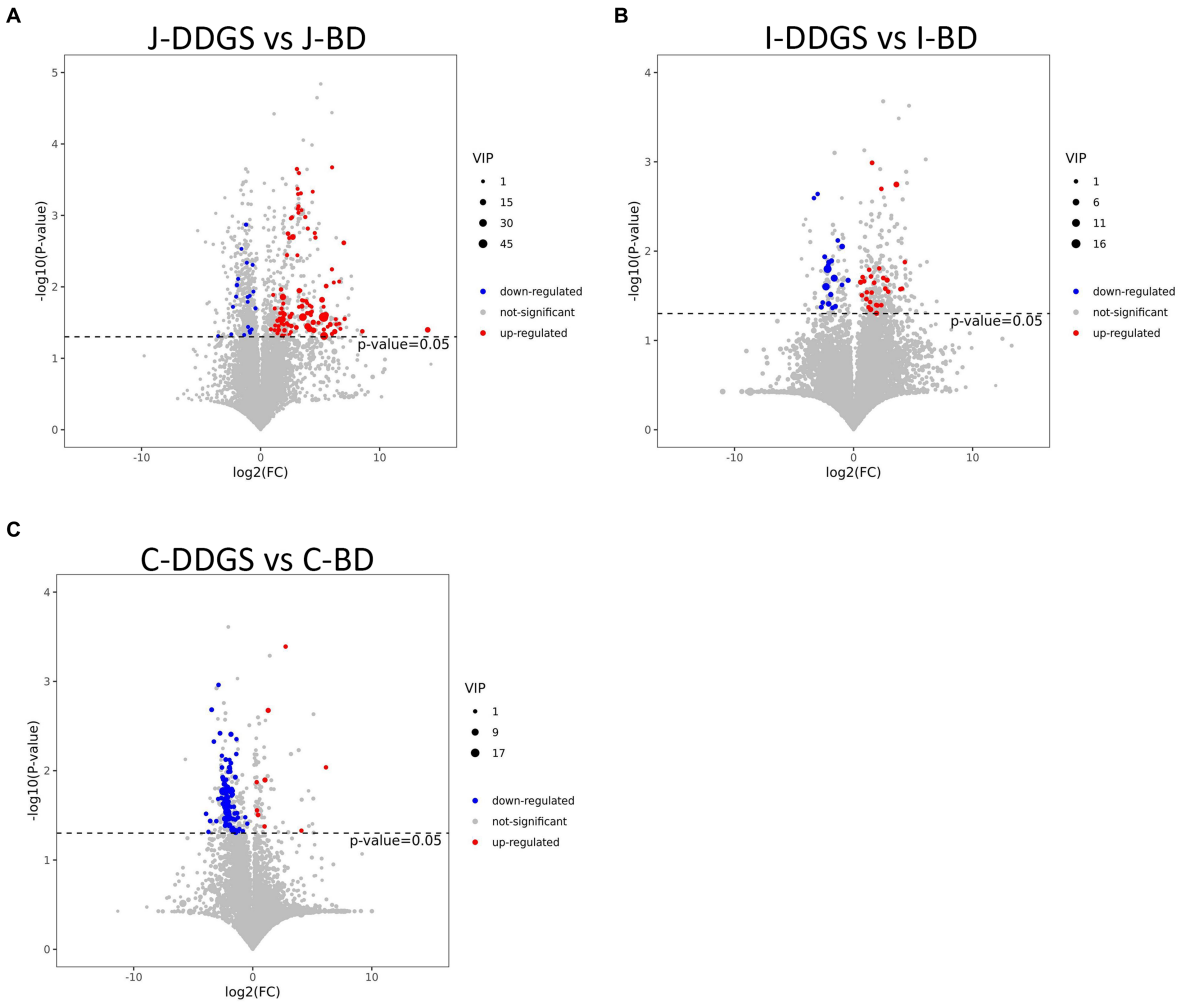
Volcano plots based on LC–MS of intestinal metabolites obtained from DDGS and BD groups. Volcano plot based on LC–MS of jejunum **(A)**, ileum **(B)**, and cecum **(C)** metabolites. The 1,399 significantly altered metabolites in the model group. Red and blue represent upregulated and downregulated metabolites, respectively, with VIP >1, fold change >1.3, and *p* < 0.05. The gray area indicates unchanged metabolites with a fold change <1.3 and *p* > 0.05. BD, basal diet group, DDGS: mixed diet group containing 25% DDGS derived from Moutai-flavored DG.

To visualize the results of the fold change (FC) analysis of 1,399 metabolites for quick identification of significantly differential metabolites, a volcano plot was drawn by transferring the FC values of each metabolite to log_2_ (FC), and by transferring the value of *p* (*p* = 0.05) of the student’s *t*-test to -log (*p*) and simultaneously meeting the variable importance in projection (VIP) values >1 for the first principal component. As shown in Figures 2A–C, a total of 123 metabolites (103 upregulated with log_2_(FC) >0 and 20 downregulated with log_2_(FC) <0), 47 metabolites (28 upregulated and 19 downregulated), and 88 metabolites (9 upregulated and 79 downregulated) were obtained in the J-DDGS group, I-DDGS and C-DDGS groups, respectively.

**Figure 3:**
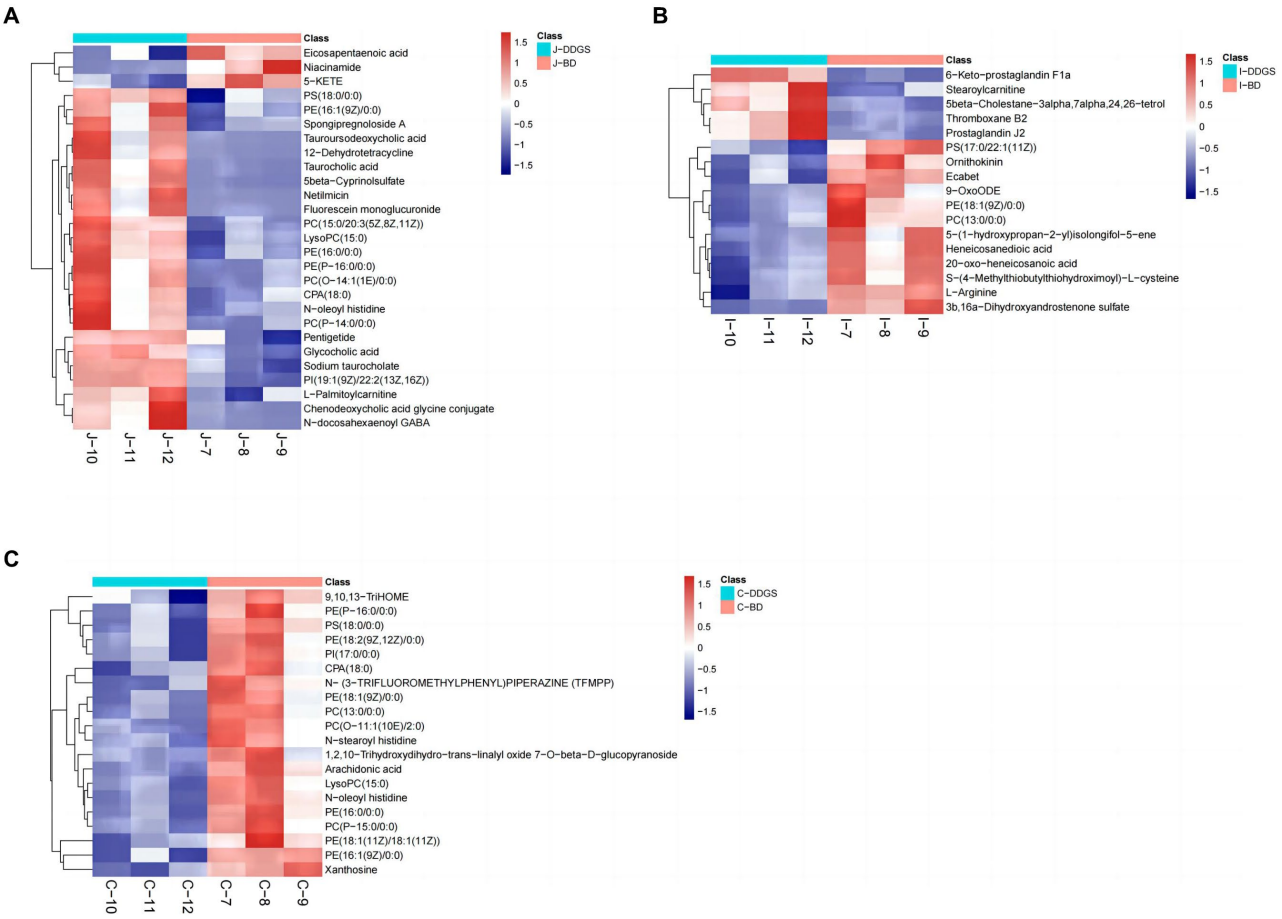
Heatmap of top 50 differential metabolites in the jejunum, ileum, and cecum. Heatmap plot based on LC-MS analyses of J-DDGS vs. J-BD **(A)**, I-DDGS vs. I-BD **(B)**, and C-DDGS vs. C-BD **(C)** comparisons, respectively. The graph displays the differential metabolites on the *Y*-axis and the sample names on the *X*-axis. The color gradient from blue to red represents the expression abundance of metabolites, with red indicating higher expression abundance and blue indicating lower expression abundance. BD, basal diet group, DDGS: mixed diet group containing 25% DDGS derived from Moutai-flavored DG.

The levels of the top 50 differential metabolites were visualized by a heat map depicted in Figures 3A–C, in which colors represent increased (red) or decreased (blue) abundance, with the intensity reflecting the corresponding concentration. Of these, the relative richness (referring to the ratio of DDGS/BD) of 23, 4, and 4 metabolites were decreased in the J-DDGS, I-DDGS, and C-DDGS groups, respectively. Conversely, the richness of 3, 12, and 11 metabolites was significantly increased in the aforementioned jejunum, ileum, and cecum comparisons, respectively. Furthermore, the relative richness of 1, 1, and 5 metabolites had no alteration in the aforementioned comparisons.

**Figure 4:**
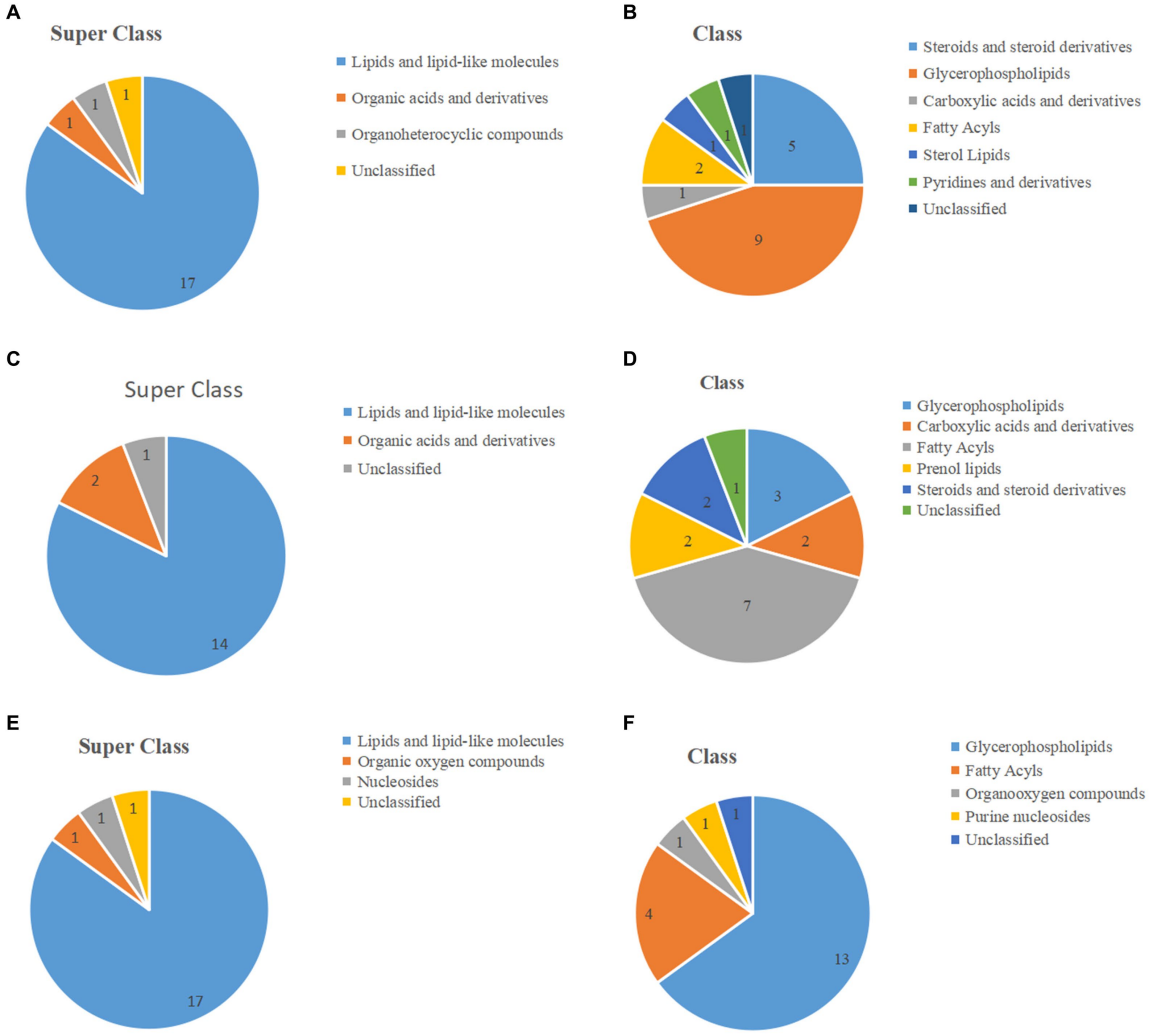
The pie chart shows the classification of the top 20 significant metabolites of the jejunum, ileum, and cecum. **(A,C,E)** The top 20 metabolites of the jejunum, ileum, and cecum are classified into superclasses. **(B,D,F)** The top 20 metabolites of the jejunum, ileum, and cecum are further divided into classes. Numbers indicate the number of each classification.

The classification of the top 20 significantly differential metabolites based on volcano plot and heat map results are shown in Figures 4A–F, with Table 1 providing detailed information. Of these, in the J-DDGS vs. J-BD comparison, they were mainly classified into lipids and lipid-like molecules (5 steroids and steroid derivatives, 9 glycerophospholipids, 2 fatty acyls, and 1 sterol lipid), organic acids and derivatives (1 carboxylic acid and derivatives), and organoheterocyclic compounds (1 pyridine and derivatives). As for the ileum, the differential metabolites of the I-DDGS vs. I-BD comparison were mainly classified into lipids and lipid-like molecules (3 glycerophospholipids, 7 fatty acyls, 2 prenol lipids and 2 steroids and steroid derivatives) and organic acids and derivatives (1 carboxylic acid and derivatives). For the cecum, the differential metabolites of the C-DDGS vs. C-BD comparison were mainly classified into lipids and lipid-like molecules (13 glycerophospholipids and 4 fatty acyls), organic oxygen compounds (1 organooxygen compound), and nucleosides (1 purine nucleosides). Collectively, these results indicate the jejunum, ileum, and cecum metabolite profiles are primarily influenced by DDGS diets.

**Table 1:** TOP 20 differential metabolites in different intestinal walls of DDGS-fed Guanling crossbred cattle.

Metabolites	Formula	m/z	Retention time (min)	Ion mode	VIP^a^	*p*-values^b^	Log_2_(FC)^c^
J-DDGS vs. J-BD							
Tauroursodeoxycholic acid	C26H45NO6S	498.2892	6.4239	Negative	32.2631	0.0486	5.3312
Chenodeoxycholic acid glycine conjugate	C26H43NO5	448.3065	6.5271	Negative	15.9113	0.0388	4.1098
LysoPC (15:0)	C23H48NO7P	480.3090	8.3475	Negative	15.2526	0.0139	1.8735
Glycocholic acid	C26H43NO6	466.3164	6.1023	Positive	10.6080\	0.0020	2.7012
Taurocholic acid	C26H45NO7S	498.2884	5.9180	Positive	10.3656	0.0152	5.1614
N-docosahexaenoyl GABA	C26H39NO3	414.3001	6.7901	Positive	8.0995	0.0406	4.4564
PE (16:0/0:0)	C21H44NO7P	452.2781	7.6887	Negative	7.0858	0.0242	1.9801
PE (P-16:0/0:0)	C21H44NO6P	436.2830	7.8675	Negative	6.3084	0.0261	2.4714
CPA (18:0)	C21H41O6P	438.2978	8.3146	Positive	5.0802	0.0492	2.0260
5beta-Cyprinolsulfate	C27H48O8S	531.2996	6.3824	Negative	4.0242	0.0098	5.5067
N-oleoyl histidine	C24H41N3O3	464.3127	7.9913	Negative	3.3079	0.0405	2.00561
Eicosapentaenoic acid	C20H30O2	303.2316	8.1219	Positive	2.8704	0.0416	−0.9064
PE (16:1(9Z)/0:0)	C21H42NO7P	450.2626	7.1999	Negative	1.8776	0.0481	1.8110
PC (O-14:1(1E)/0:0)	C22H46NO6P	450.2983	7.4406	Negative	1.8724	0.0238	2.6164
Spongipregnoloside A	C33H52O11	623.3431	7.7093	Negative	1.7392	0.0354	1.5054
Sodium taurocholate	C26H44NNaO7S	582.2721	5.7792	Negative	1.6224	0.0036	2.2189
PI (19:1(9Z)/22:2(13Z, 16Z))	C50H91O13P	951.5941	5.9168	Negative	1.5179	0.0007	3.1835
Niacinamide	C6H6N2O	123.0549	0.9748	Positive	1.5125	0.0394	−0.7562
PS (18:0/0:0)	C24H48NO9P	546.2809	7.8262	Negative	1.4836	0.0348	1.2241
PC (15:0/20:3(5Z, 8Z, 11Z))	C43H80NO8P	814.5612	7.7025	Negative	1.4395	0.0129	1.0551
I-DDGS vs. I-BD							
PE (18:1(9Z)/0:0)	C23H46NO7P	480.3085	8.2732	Positive	8.9231	0.0250	−2.3181
Ornithokinin	C48H77N15O11	520.8031	4.2282	Positive	7.3695	0.0201	−1.6263
6-Keto-prostaglandin F1a	C20H34O6	369.2278	5.4216	Negative	3.5467	0.0018	3.5957
L-Arginine	C6H14N4O2	175.1186	0.6238	Positive	3.1896	0.0089	−0.9584
PC (13:0/0:0)	C21H44NO7P	454.2928	8.1150	Positive	3.1868	0.0389	−2.0910
Stearoylcarnitine	C25H49NO4	428.3734	7.9795	Positive	2.3794	0.0450	1.4324
PS (17:0/22:1(11Z))	C45H86NO10P	830.5913	11.5799	Negative	2.2683	0.0212	−0.4616
20-oxo-heneicosanoic acid	C21H40O3	341.3044	8.8580	Positive	2.1972	0.0128	−1.8547
9-OxoODE	C18H30O3	277.2159	8.0256	Positive	1.8338	0.0416	−1.5293
Heneicosanedioic acid	C21H40O4	339.2893	8.2732	Positive	1.6711	0.0118	−2.4269
Thromboxane B2	C20H34O6	353.2322	5.5398	Positive	1.4171	0.0263	2.6628
5-(1-hydroxypropan-2-yl) isolongifol-5-ene	C18H30O	263.2368	9.0485	Positive	1.3218	0.0376	−2.5925
S-(4-Methylthiobutylthiohydroximoyl)-L-cysteine	C8H16N2O3S2	235.0564	0.6031	Positive	1.2965	0.0238	−0.9712
Ecabet	C20H28O5S	379.1577	8.7462	Negative	1.2668	0.0075	−1.3262
5beta-Cholestane-3alpha,7alpha,24,26-tetrol	C27H48O4	454.3891	8.0256	Positive	1.2047	0.0192	1.4576
Prostaglandin J2	C20H30O4	317.2107	5.5398	Postive	1.1707	0.0284	2.9054
3b,16a-Dihydroxyandrostenone sulfate	C19H28O6S	385.1674	9.1105	Positive	1.0291	0.0025	−3.3290
C-DDGS vs. C-BD							
LysoPC (15:0)	C23H48NO7P	480.3090	8.3475	Negative	15.1675	0.0169	−2.4709
PE (18:1(9Z)/0:0)	C23H46NO7P	480.3085	8.2732	Positive	12.6200	0.0337	−2.0644
PE (16:0/0:0)	C21H44NO7P	452.2781	7.6887	Negative	5.7268	0.0171	−2.4957
PE (P-16:0/0:0)	C21H44NO6P	436.2829	7.8675	Negative	4.7338	0.0407	−1.9767
PC (P-15:0/0:0)	C23H48NO6P	464.3139	8.5193	Negative	4.6287	0.0267	−2.2409
PC (13:0/0:0)	C21H44NO7P	454.2928	8.1150	Positive	4.4824	0.0256	−2.3107
CPA (18:0)	C21H41O6P	438.2978	8.3146	Positive	3.5690	0.0490	−1.4364
PE (18:2(9Z,12Z)/0:0)	C23H44NO7P	476.2782	7.3993	Negative	2.9291	0.0456	−1.7521
N-oleoyl histidine	C24H41N3O3	464.3127	7.9913	Negative	2.6850	0.0092	−1.9593
PS (18:0/0:0)	C24H48NO9P	546.2809	7.8262	Negative	2.0920	0.0153	−2.1028
PC (O-11:1(10E)/2:0)	C21H42NO7P	452.2776	7.9657	Positive	1.7919	0.0202	−2.6395
PE (16:1(9Z)/0:0)	C21H42NO7P	450.2625	7.1998	Negative	1.7826	0.0102	−1.8965
N-stearoyl histidine	C24H43N3O3	466.3275	8.5674	Negative	1.7181	0.0234	−2.5048
PI (17:0/0:0)	C26H51O12P	631.3092	8.3405	Negative	1.7167	0.0198	−2.0960
1,2,10-Trihydroxydihydro-trans-linalyl oxide 7-O-beta-D-glucopyranoside	C16H30O10	403.1578	8.6431	Negative	1.5055	0.0497	−1.5341
Arachidonic acid	C20H32O2	303.2323	8.6431	Negative	1.3587	0.0065	−1.3843
PE (18:1(11Z)/18:1(11Z))	C41H78NO8P	742.5400	6.0179	Negative	1.1811	0.0391	−0.4565
Xanthosine	C10H12N4O6	283.0678	2.5247	Negative	1.1507	0.0044	−1.3721
9,10,13-TriHOME	C18H34O5	329.2331	6.0797	Negative	1.0758	0.0475	−0.8254
N-(3-trifluoromethylphenyl)piperazine (TFMPP)	C11H13F3N2	251.0776	2.35975	Negative	1.0406	0.0207	−2.9114

BD, basal diet group, DDGS: mixed diet group containing 25% DDGS derived from Moutai-flavored DG. J, jejunum; I, ileum; C, cecum. ^a^VIP was obtained from the OPLS-DA model, ^b^*p* was calculated by student’s *t*-test analysis, *p* < 0.05, ^c^Log_2_(FC) indicates the ratio of the average expression of metabolites in the two groups of samples, with positive values indicating upregulation and negative values indicating downregulation.

### 3.3 KEGG pathway analysis

**Figure 5:**
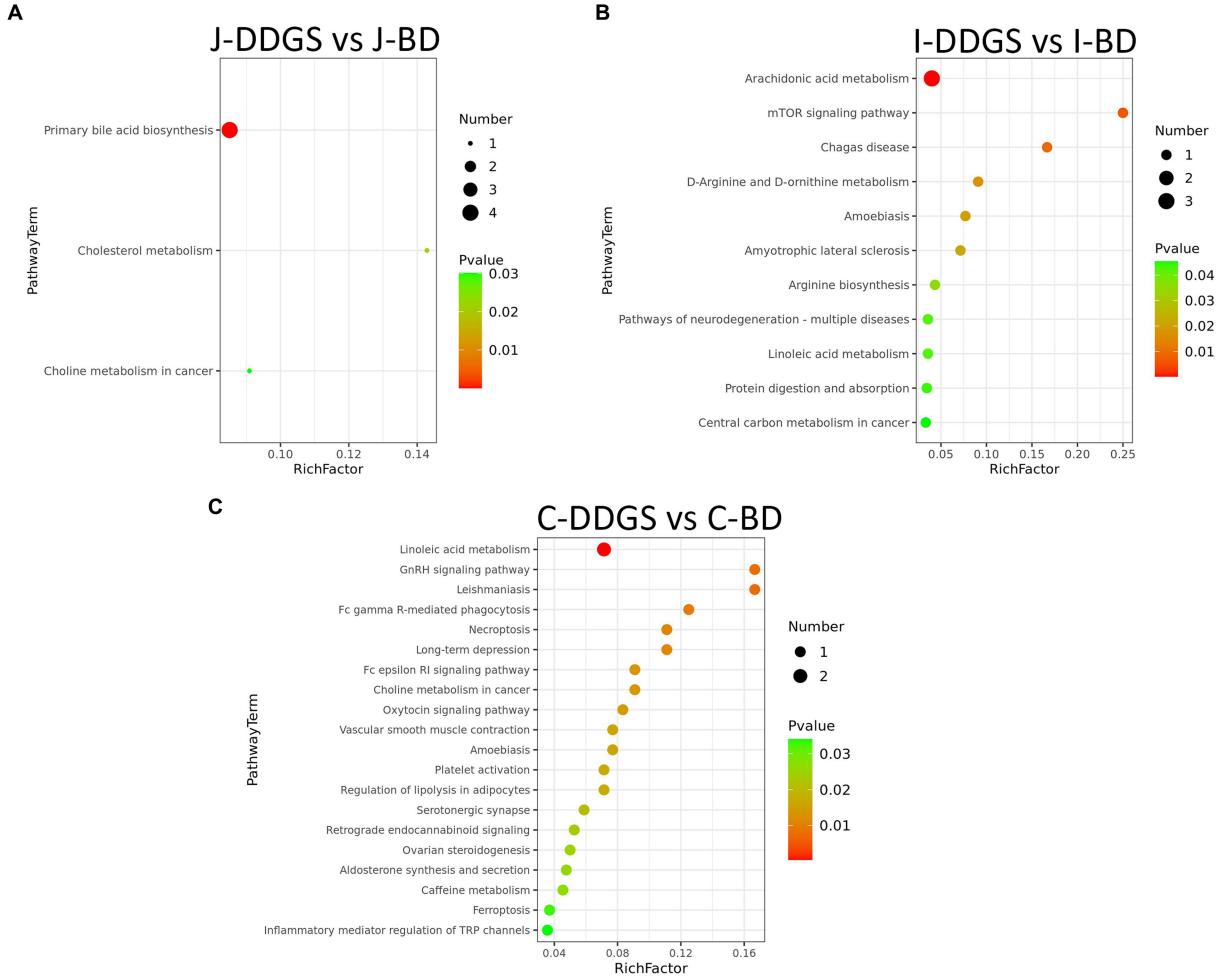
Analysis of the KEGG pathway. Pathway analysis of differentially expressed intestinal metabolites profiles in J-DDGS vs. J-BD **(A)**, I-DDGS vs. I-BD **(B)**, and C-DDGS vs. C-BD comparisons **(C)**. The *X*-axis represents pathway impact and the *Y*-axis represents the pathway enrichment. The larger size of the circle indicates greater pathway enrichment and the darker color indicates higher pathway impact values. The closer the color is to red, the smaller the value of *p* is. Significantly different metabolic pathways are marked around each dot with differential metabolites. BD, basal diet group, DDGS: mixed diet group containing 25% DDGS derived from Moutai-flavored DG.

Next, we investigated which metabolic pathways may be behind the observed intestinal metabolic profile changes associated with DDGS diets. The identified differential metabolites were annotated with KEGG and HMDB. In this study, a total of 34 significant metabolic pathways were identified (*p* < 0.05, −log(*p*) > 1.3). Among them, there were 3 significant pathways, namely, primary bile acid biosynthesis, cholesterol metabolism, and choline metabolism in cancer, that were significantly enriched in the J-DDGS group; there were 11 significant pathways (including arachidonic acid metabolism, mTOR signaling pathway, and D-arginine and D-ornithine metabolism, etc.) that were significantly enriched in the I-DDGS group; and there were 20 significant pathways (including linoleic acid metabolism, GnRH signaling pathway, and leishmaniasis, etc.) that were significantly enriched in the C-DDGS group (Figures 5A–C). The detailed information is shown in Table 2. Furthermore, three metabolic pathways, namely, primary bile acid biosynthesis, linoleic acid metabolism, and arachidonic acid metabolism, were found to be linked to intestinal inflammation and immunity. Among them, the primary bile acid biosynthesis and arachidonic acid metabolism metabolic pathways were specific to the jejunum and ileum, respectively. While the linoleic acid metabolism metabolic pathway was shared in the ileum and cecum.

**Table 2:** The list of metabolic pathways with significant differences in the DDGS group compared to the BD group.

ID annotation	Annotation	In set	−log(*p*)	FDR	*p*-values
J-DDGS vs. J-BD					
bta00120	Primary bile acid biosynthesis	4	5.3348	0.0000	0.000
bta04979	Cholesterol metabolism	1	1.7150	0.1060	0.0193
bta05231	Choline metabolism in cancer	1	1.5208	0.1106	0.0301
I-DDGS vs. I-BD					
bta00590	Arachidonic acid metabolism	3	3.9398	0.0016	0.0001
bta04150	mTOR signaling pathway	1	2.2109	0.0430	0.0062
bta05142	Chagas disease	1	2.0353	0.0430	0.0092
bta00472	D-arginine and D-ornithine metabolism	1	1.7734	0.0499	0.0169
bta05146	Amoebiasis	1	1.7014	0.0499	0.0199
bta05014	Amyotrophic lateral sclerosis	1	1.6695	0.0499	0.0214
bta00220	Arginine biosynthesis	1	1.4563	0.0578	0.0350
bta05022	Pathways of neurodegeneration – multiple diseases	1	1.3722	0.0578	0.0424
bta00591	Linoleic acid metabolism	1	1.3722	0.0578	0.0424
bta04974	Protein digestion and absorption	1	1.3572	0.0578	0.0440
bta05230	Central carbon metabolism in cancer	1	1.3428	0.0578	0.0454
C-DDGS vs. C-BD					
bta00591	Linoleic acid metabolism	2	3.3700	0.0317	0.0004
bta04912	GnRH signaling pathway	1	2.1319	0.0317	0.0074
bta05140	Leishmaniasis	1	2.1319	0.0317	0.0074
bta04666	Fc gamma R-mediated phagocytosis	1	2.0073	0.0317	0.0098
bta04217	Necroptosis	1	1.9564	0.0317	0.0111
bta04730	Long-term depression	1	1.9564	0.0317	0.0111
bta04664	Fc epsilon RI signaling pathway	1	1.8696	0.0317	0.0135
bta05231	Choline metabolism in cancer	1	1.8696	0.0317	0.0135
bta04921	Oxytocin signaling pathway	1	1.8321	0.0317	0.0147
bta04270	Vascular smooth muscle contraction	1	1.7975	0.0317	0.0159
bta05146	Amoebiasis	1	1.7975	0.0317	0.0159
bta04611	Platelet activation	1	1.7655	0.0317	0.0172
bta04923	Regulation of lipolysis in adipocytes	1	1.7655	0.0357	0.0172
bta04726	Serotonergic synapse	1	1.6818	0.0358	0.0208
bta04723	Retrograde endocannabinoid signaling	1	1.6339	0.0358	0.0232
bta04913	Ovarian steroidogenesis	1	1.6118	0.0358	0.0244
bta04925	Aldosterone synthesis and secretion	1	1.5908	0.0358	0.0257
bta00232	Caffeine metabolism	1	1.5708	0.0409	0.0269
bta04216	Ferroptosis	1	1.4829	0.0409	0.0330
bta04750	Inflammatory mediator regulation of TRP channels	1	1.4673	0.0317	0.0341

Based on the selection threshold of −log(*p*) > 1.3 and *p* < 0.05, 3 significant target pathways were obtained. In set means the number of differential metabolites involved in this pathway; the *p*-value is calculated from the enrichment analysis; FDR value is the false discovery rate to adjust the *p*-values.

## 4 Discussion

Kweichow Moutai liquor is a world-famous Jiang-flavor Baijiu with excellent and numerous fermentation microorganisms. It produces large numbers of nutrient-rich DGs (18). Improper disposal of fresh DGs easily causes waste of resources and environmental pollution, including soil contamination. Reasonable reuse of DGs as feed resources in livestock production is necessary (19). Drying DGs to obtain DDGS is a common strategy for reuse of DGs. Studies have shown that feeding dried DGs can effectively improve the growth performance, slaughter performance, and intestinal development of bulls and other ruminants, making DDGS suitable for available feed resources (20–22). Dried DGs as animal feed additives do not cause alcohol intoxication in animals. Much of the current research revolves around the effect of feeding DDGS diets on the structural metabolism of gut flora in pigs and small animals (23–28). Nevertheless, there is a paucity of reports on the effect of feeding DDGS diets on gut tissue metabolism in ruminants such as cattle. In this study, it was found that the intestinal metabolomic profile of Guanling crossbred cattle fed a DDGS diet differed significantly from those fed a BD diet. The DDGS diet was shown to influence several intestinal metabolic pathways, such as primary bile acid biosynthesis, linoleic acid metabolism, and arachidonic acid metabolism. These pathways are known to be linked to intestinal immunity and inflammation. This implies that the DDGS diet has the potential to positively function in enhancing immunity and improving intestinal inflammation in cattle.

Primary bile acid biosynthesis is a metabolic pathway that is significantly enriched in the jejunum of the DDGS group. Notable metabolites in this pathway include 5beta-cyprinolsulfate, chenodeoxycholic acid glycine conjugate, glycocholic acid, and taurocholic acid, all of which showed significant upregulation in the J-DDGS group (Figure 5A). During primary bile acid biosynthesis, bile acid is synthesized in the liver using bile acid synthase (CYP7A1). This process forms glycocholic acid with glycine, which facilitates the digestion and absorption of fats by emulsifying them and dispersing them in aqueous solution (29, 30). Taurocholic acid, resulting from the fusion of bile acid and taurine, is capable of linking with fats and creating bile salt-fatty acid complexes. This, in turn, promotes the breakdown and absorption of fat while also halting fat build-up in the intestine (31). Synthesized by the enzyme-catalyzed reaction of normal bile acids with sulfuric acid, 5-beta-cyprinolsulphate is a bioactive component of fish oil with antioxidant and anti-inflammatory properties, as well as being capable of regulating the balance of intestinal flora (32). Chenodeoxycholic acid-glycine conjugate is formed by the combination of bile ketone acid (produced by the carboxylation of bile acids in the liver) and glycine, which promotes bile secretion and thus increases fat emulsification and absorption (33, 34). This study and a previously published manuscript (18) suggest that the DDGS diet may promote intestinal fat emulsification and absorption by upregulating the levels of 5beta-cyprinolsulfate, chenodeoxycholic acid-glycine conjugate, glycocholic acid, and taurocholic acid, and by reducing the number of harmful bacteria and increasing the number of beneficial bacteria, thereby improving intestinal health. However, more research is needed to understand these effects.

Arachidonic acid metabolism is ileum-specific, and 6-Keto-prostaglandin F1a, prostaglandin J2, and thromboxane B2 (prostaglandin analogs) are end-products of this pathway, which were markedly upregulated in the I-DDGS group (Figure 5B). 6-Keto-prostaglandin F1a is formed through the metabolic conversion of PGE1 *in vivo*, which reduces intestinal mucosal damage and relieves gastrointestinal ulcers, as well as having anti-platelet aggregation and anti-inflammatory effects (35–37). Prostaglandin J2 is a substance that is enzymatically converted from prostaglandin H2 (PGH2) or other prostaglandin components by enzymatic action and can promote apoptosis by regulating the production of inflammatory mediators (38, 39). Thromboxane B2 is a platelet-aggregating hormone, a stable metabolite of thromboxane A2 produced through the catalytic reaction of cyclooxygenase and thromboxane synthetase, which has the effect of regulating blood flow, maintaining vascular function, and platelet aggregation (40, 41). Our study showed that feeding DDGS diets exerted a protective effect on the intestine by upregulating the levels of 6-Keto-prostaglandin F1a and prostaglandin J2, which helped maintain the integrity and health of the intestinal mucosa and inhibited intestinal inflammation and tumor cell growth. Upregulation of thromboxane B2 levels may have a negative effect on intestinal blood flow and vascular function. However, more research is needed to understand its specific effects.

Linoleic acid metabolism is shared by the ileum and cecum. 9-OxoODE, 9,10,13-TriHOME, and arachidonic acid are all secondary metabolites in the linoleic acid metabolism, significantly downregulated in I-DDGS and C-DDGS groups. Linoleic acid metabolites are involved in regulating several aspects of the inflammatory process. Among them, 9-OxoODE and 9,10,13-TriHOME are pro-inflammatory and are produced through lipoxygenase-catalyzed linoleic acid metabolism, which can influence the production of inflammatory mediators, such as pro-inflammatory cytokines and leukocyte chemokines (42, 43). Arachidonic acid is a polyunsaturated fatty acid, which is metabolized mainly through the tapping pathway enzymes that catalyze cyclooxygenase and lipoxygenase into metabolites with a variety of biological activities; these metabolites are involved in the genesis and regulation of intestinal inflammation, and reducing their production helps to inhibit the inflammatory process (44, 45). Our study demonstrated that DDGS diets were able to attenuate intestinal inflammation and reduce inflammatory mediators by downregulating the concentrations of 9-OxoODE, 9,10,13-TriHOME, and arachidonic acid, thereby reducing the severity of the inflammatory response. Notably, the effects of linoleic acid metabolites may vary depending on the type and concentration of the particular compound. Further studies are needed to more accurately understand the specific mechanisms of action of linoleic acid metabolites in intestinal inflammation and immunomodulation.

## 5 Conclusion

Taken together, our findings suggest that implementing a DDGS diet can encourage the production of primary bile acids and promote arachidonic acid metabolism. This is achieved by increasing levels of 5beta-cyprinolsulfate, chenodeoxycholic acid glycine conjugate, glycocholic acid, and taurocholic acid, as well as 6-Keto-prostaglandin F1a, prostaglandin J2, and thromboxane B2 (prostaglandin analogs). Additionally, the levels of 9-OxoODE, 9,10,13-TriHOME, and arachidonic acid inhibition of linoleic acid metabolism are reduced by this diet. The equilibrium of three metabolic pathways sustains a proportional level of intestinal inflammation and immune function, fostering gut inflammation and immunity in Guanling crossbred cattle. Nonetheless, this is solely a preliminary investigation, and extensive analysis with a vast number of animals is necessary to verify the particular regulatory roles and their underlying mechanisms. This study enhances our comprehension of the nutritional and metabolic effects of DDGS diets on Guanling crossbred cattle and sheds light on their underlying mechanisms. Additionally, it provides valuable references for the use of DDGS as a feed for animals.

## Data availability statement

The original contributions presented in the study are included in the article/Supplementary material, further inquiries can be directed to the corresponding authors.

## Ethics statement

The animal studies were approved by EAE-GZu-2020-E018, 2 September 2020. The studies were conducted in accordance with the local legislation and institutional requirements. Written informed consent was obtained from the owners for the participation of their animals in this study.

## Author contributions

XL, EZ, ZeC, and CC conceived the study. XL, TZ, DX, and MZ performed the experiments. XL, JZ, RZ, GH, EZ, ZhC, and CC analyzed experimental results and data. GH, BZ, KW, CC, and SM assisted with animal experiments. XL wrote the manuscript. All authors have read and agreed to the published version of the manuscript.

## Footnotes

^1^ http://www.kegg.jp/kegg/pathway.html
