# Supplementary material for: Effect of feeding a dried distillers’ grains with solubles diet on the metabolism of the intestinal wall in Guanling crossbred cattle: a preliminary assessment
Source: Front Vet Sci. 2024 Jan 9;10:1223088. doi: 10.3389/fvets.2023.1223088 (PMC10803422; doi:10.3389/fvets.2023.1223088)
Supplement: Supplementary file 1 [file Data_Sheet_1.doc]

Supplementary Table:

**Table S1 Experimental concentrate supplement formulations (DM) %**

| Item | BD  Group | DDGS  Group |
| --- | --- | --- |
| DDGS | 0 | 25.0 |
| GC | 52.1 | 42.0 |
| WB | 17.2 | 10.0 |
| SM | 16.0 | 10.8 |
| RM | 10.8 | 8.30 |
| Premixes | 0.10 | 0.10 |
| salt | 1.00 | 1.00 |
| CB | 0.50 | 0.50 |
| SB | 1.00 | 1.00 |
| RF | 1.20 | 1.20 |
| MR | 0.10 | 0.10 |
| Total | 100 | 100 |

Note: DM: Dry matter base; BD: a basal diet group; DDGS: a mixed diet group containing 25% DDGS derived from Moutai-flavored DG; GC: Ground corn; WB: wheat bran; SM: soybean meal; RM: rapeseed meal; CB: calcium bisulfate; SB: Sodium bicarbonate; RF: rock fine; MR: Mold Remover.

**Table S2 The feed energy of diets in the BD group and DDGS group**

| Item | BD Group concentrates | DDGS Group  concentrates | *Pennisetum* *Sinese*  Roxb |
| --- | --- | --- | --- |
| Feed energy level, % |  |  |  |
| Dry matter | 87.35 | 87.77 | 46.95 |
| Gross energy (MJ/kg) | 17.23 | 17.06 | 16.39 |
| Crude protein | 17.65 | 17.94 | 4.37 |
| Neutral detergent fiber | 28.20 | 28.58 | 69.74 |
| Acid detergent fiber | 5.47 | 5.25 | 45.35 |
| Total P | 0.32 | 0.35 | 0.78 |
| Total K | 0.68 | 0.67 | 1.37 |
| Calcium | 0.74 | 0.76 | 0.59 |

**Table S3 Chromatographic gradient Conditions for UPLC-Q-TOF/MS analysis**

| Time (min) | Flow rate  (mL /min) | Mobile phase A (%) | Mobile phase B (%) |
| --- | --- | --- | --- |
| 0 | 0.4 | 99 | 1 |
| 1 | 0.4 | 70 | 30 |
| 2.5 | 0.4 | 40 | 60 |
| 6.5 | 0.4 | 10 | 90 |
| 8.5 | 0.4 | 0 | 100 |
| 10.7 | 0.4 | 0 | 100 |
| 10.8 | 0.4 | 99 | 1 |
| 13 | 0.4 | 99 | 1 |

**Table S4 Chromatographic gradient Conditions for UPLC-Q-TOF/MS analysis**

| MS conditions | |
| --- | --- |
| Capillary voltages | 2.5 kV（ES+）2.5 kV（ES-） |
| Declustering potential | 40 V  4 eV  115 ℃  450 m  900 L/h  50-1000 amu  0.2 s  0.02 s |
| Collision energy |
| Source temperature |
| Desolvation temperature |
| Desolvation gas flow |
| Mass range |
| Scan time |
| interscan delay |

**Supplementary Figures:**

**Figure S1**


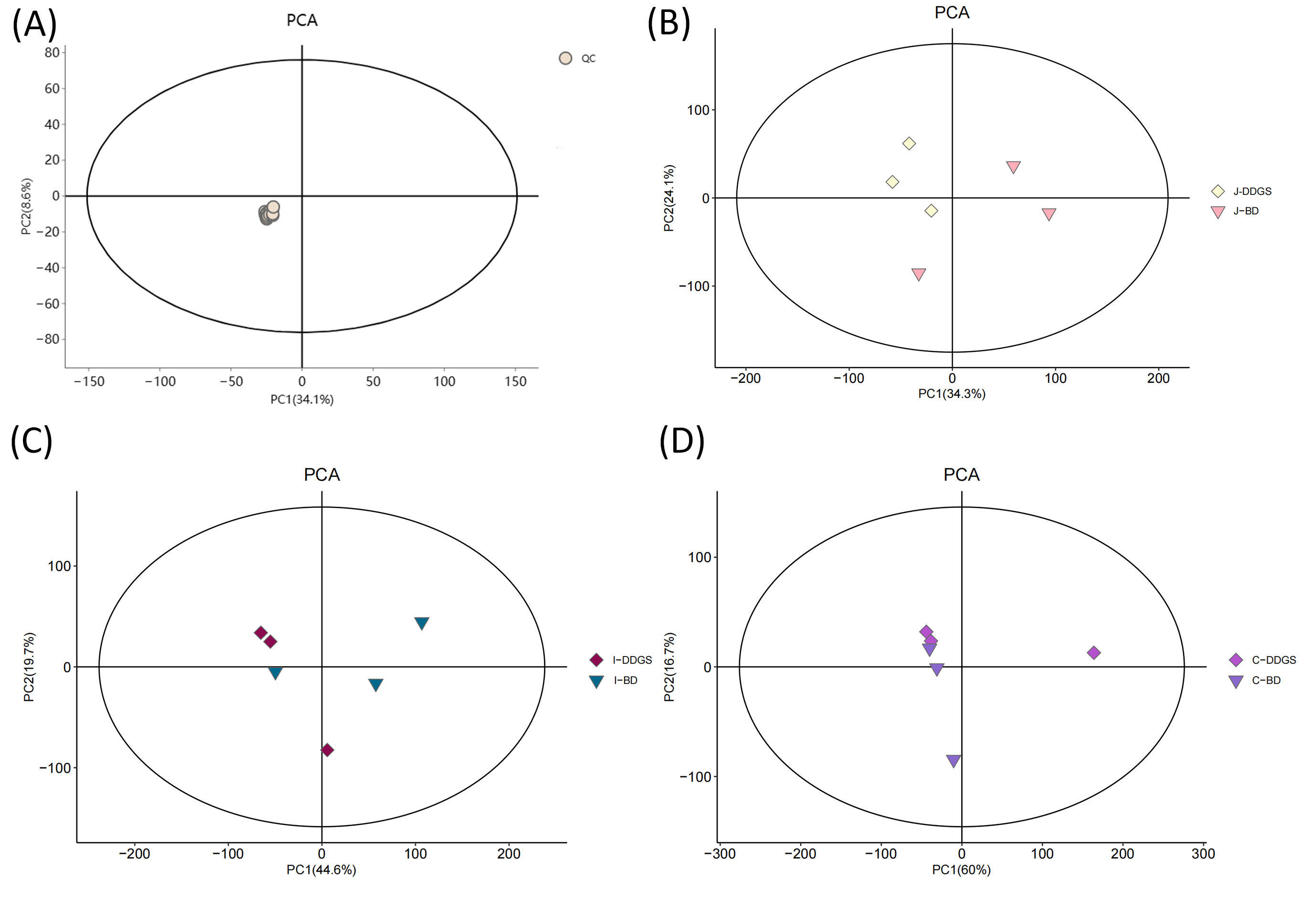


**Figure 1 PCA for data distribution.** (A) QC group, (B) J-DDGS B vs J-BD comparison, (C) I-DDGS vs I-BD comparison, (D) C-DDGS vs C-BD comparison. Each dot, triangle, or diamond on the plot represents a sample in the corresponding group. Abbreviation: BD, a basal diet group, DDGS: a mixed diet group containing 25% DDGS derived from Moutai-flavored DG.
